# Supplementary material for: Are carbon emissions trading and green financial instruments synergistic? -Comprehensive quantitative research based on content analysis
Source: PLoS One. 2024 Mar 7;19(3):e0298601. doi: 10.1371/journal.pone.0298601 (PMC10919601; doi:10.1371/journal.pone.0298601)
Supplement: S1 Data — (ZIP) [file pone.0298601.s001.zip › Data/地级市Z_F工作报告.zip!/╜¡╦╒/╦╒╓▌╩╨/└┤╫╘╣1⁄2╓┌║┼í╛┬φ┐╦╩2╛▌═°í┐_2018.docx]

各位代表：

　　现在，我代表苏州市人民政府，向大会报告工作，请予审议，并请市政协各位委员和其他列席人员提出意见。

　　一、2017年工作回顾

　　过去的一年，我们以迎接党的十九大召开、学习宣传贯彻党的十九大精神为强大动力，在中共苏州市委的领导下，全面落实中央和省委、省政府的决策部署，坚持稳中求进工作总基调，积极开展“两聚一高”新实践，团结依靠全市人民，统筹做好稳定增长、创新驱动、改革开放、城乡建设、生态优化、民生改善等工作，推动经济社会持续发展，较好完成了市十六届人大一次会议确定的主要目标任务。

　　1．经济保持平稳增长，质量效益逐步提高。全市预计完成地区生产总值1.7万亿元，增长7%。实现一般公共预算收入1908.1亿元，增长10.3%。工业经济持续向好，实现规模以上工业总产值3.2万亿元，增长10.4%；工业增加值率提高0.5个百分点，工业全员劳动生产率增长12%，利税、利润分别增长18%和25%。投资结构继续优化，完成全社会固定资产投资5600亿元，服务业投资占到64.4%，民间投资占到60.7%。市场消费增长较快，实现社会消费品零售总额5430亿元，增长10%；旅游总收入增长12%，我市获评首批中国休闲旅游示范城市；文化创意产业营业收入增长12%，我市入选全国文化消费试点城市。供给侧结构性改革取得实效，关停淘汰落后低效企业996家，压减钢铁产能83万吨、平板玻璃产能330万重量箱；房地产市场保持平稳，商业办公用房库存逐步下降；新增上市公司14家，上市公司首发融资及再融资327亿元；降低企业成本480亿元；114个补短板项目完成投资超过260亿元。

　　2．科技创新成效明显，产业转型步伐加快。落实研发费用加计扣除等政策，推动企业研发投入增长10%，全社会研究与试验发展经费支出占地区生产总值比重预计达到2.82%。纳米真空互联实验站、干细胞实验平台、中国移动研发中心等重大创新载体建设取得明显进展，中科院上海技术物理所苏州研究院、北京大学分子工程苏南研究院、北京航空航天大学苏州创新研究院、哈佛大学韦茨创新中心、省产业技术研究院阳澄国际研发产业园等落户苏州。设立创新产业发展引导基金，发放“科贷通”贷款70亿元，全市创投机构管理资金1203亿元。新增高层次人才2.25万人、高技能人才2.2万人；新增国家“千人计划”人才18人，总数达到237人。获批设立中国苏州国际人力资源服务产业园。成功举办第九届国际精英创业周、2017年中国国际技能大赛苏州赛区赛事。知识产权强市加快建设，万人有效发明专利拥有量增加7.2件，达到45.5件；苏州知识产权法庭获准成立，江苏国际知识产权运营交易中心投入运营，我市成为国家知识产权运营服务体系建设重点城市。

　　推动产业提档升级，经济结构不断优化。新兴产业产值、高新技术产业产值占规模以上工业总产值比重分别提高1个和0.9个百分点。新认定国家高新技术企业1573家，获评“十三五”首批省级先进制造业基地2家，新增省级示范智能车间52个，昆山小核酸及生物医药产业基地获批国家火炬计划特色产业基地。建成工业企业资源集约利用大数据平台，对规模以上工业企业和实际用地3亩以上的规模以下工业企业实施综合评价。“中国制造2025”国家级示范区创建现场会在我市召开。预计全市服务业增加值增长8.3%。获评首批省级生产性服务业集聚示范区3家。昆山市陆家镇、常熟市海虞镇、吴江区七都镇入选第二批全国特色小镇。农业现代化步伐加快，新增现代农业园区面积7万亩、高标准农田6.2万亩、高效设施农（渔）业1万亩，新增新型职业农民1400人。成功举办首届全国新农民新技术创业创新博览会。高质量完成第三次全国农业普查主体工作。

　　3．各项改革积极推进，开放优势巩固提升。“放管服”改革不断深化，下放行政审批事项89项；市场监管信息平台上线运行，事中事后监管得到加强。相对集中行政许可权改革试点深入实施，苏州市和张家港市、昆山经济技术开发区、张家港保税区行政审批局挂牌成立。企业“多证合一、一照一码”、个体工商户“两证合一”和不动产交易登记“一窗受理、集成服务”等改革措施全面推行，企业登记全程电子化试点加快推进。新增私营企业10.6万家、注册资本6071亿元。国有企业分类管理与考核、负责人薪酬制度改革积极实施，苏州资产管理有限公司开业运行。政府投资项目代建制加快推行。跨境人民币创新业务、跨境融资宏观审慎管理政策成效明显。土地承包经营权有偿退出、以农村社区为基本单位的村民自治、政府购买农业公益性服务机制创新、重要农产品收入保险、农村集体资产股份权能改革等国家级农村改革试点工作全面开展，股权固化改革试点任务基本完成，土地承包经营权确权颁证率超过97%，农村产权交易分中心实现县级市、区全覆盖，农村集体资产资源资金监管不断强化。

　　创新对外开放体制机制，开放型经济发展取得新成果。苏州工业园区在国家级经济技术开发区综合发展水平考核中名列第一，14项开放创新举措向全国推广；昆山市获批建设对台科技合作与交流基地；太仓市中德智能制造合作创新园入选国家工信部试点示范项目。跨境电子商务、服务贸易、市场采购贸易、综合保税区内企业增值税一般纳税人资格等开放创新试点进展顺利。全市实现进出口总额3130亿美元，增长14.3%，其中出口1846亿美元，增长12.6%。实际使用外资60.6亿美元，新兴产业和高技术项目使用外资占到51.2%。对“一带一路”沿线国家和地区的进出口增长14.8%，协议投资额增长85%。太仓港完成集装箱吞吐量451万标箱，增长10.5%。外事、对台、侨务工作全面加强，成功举办2017世界城市峰会、中国—东盟新闻部长会议和第八届中国—东盟知识产权局长会议。南北挂钩、对口支援和帮扶工作积极开展，东西扶贫协作不断深化。

　　4．城乡发展协调并进，环境面貌有效改善。认真开展苏州市城市总体规划（2035）编制和综合交通体系规划修编工作，我市成为国家新一轮城市总体规划编制改革试点城市和首批城市设计试点城市。调整完善市、县级市土地利用总体规划，永久基本农田和城镇开发边界全面划定。沪通铁路苏州段建设进展顺利，通苏嘉、沪苏湖、太仓港港口支线等铁路前期工作抓紧开展。沪宁高速公路昆山高新区互通、吴淞江大桥和西环快速路南延主线高架段顺利建成。轨道交通4号线及支线开通运营，3号线和5号线加快建设。城北路、澄阳路、太湖新城地下综合管廊基本建成，海绵城市试点项目稳步推进。新增人防设施130万平方米。我市成为全国生态修复、城市修补试点城市和5G通信试点城市。城乡环境综合整治专项行动接续开展，历史文化名城保护和提升六大工程积极实施，铁路、高速公路和内河主航道沿线绿化及环境整治工作基本完成，新建改建公共卫生间251座，新增城乡生活垃圾分类试点200个。农村集体总资产达到1840亿元，增长7%，村均年稳定性收入达到815万元。新建成10个康居特色村和350个三星级康居乡村，5个特色田园乡村进入首批省级试点，吴中区临湖镇石舍村获评全国美丽乡村示范村，太仓市沙溪镇、吴中区甪直镇成为国家园林城镇。

　　加大环境保护力度，生态文明建设成效显著。狠抓中央、省环保督察反馈问题整改，大力开展“两减六治三提升”和沿江化工行业优化提升整治专项行动，完成年度减煤目标任务，淘汰落后化工企业174家，整治完成黑臭河道54条，关停规模化畜禽养殖场320家，实施984家企业挥发性有机物治理和614家建筑工地扬尘整治，新增危险废物年焚烧处置能力4.5万吨。实施大气污染防治工程，市区空气质量优良天数达到261天，比上年增加9天，PM2.5平均浓度下降6.5%。太湖流域水污染防治和阳澄湖生态优化行动深入开展，四级河长体系全面建立，巡河、治河、护河工作积极推进，国考、省考断面水质全部达标，其中优Ⅲ比例分别达到75%和74%。农村生活污水治理三年行动计划顺利收官，治理率超过80%；城市中心区清水工程加快建设。我市通过国家水生态文明城市建设试点验收。制定土壤污染防治工作方案，设置国控省控土壤监测点位130个，涉重金属企业遗留地块排查工作如期完成。加强城乡绿化，市区新增绿地400万平方米，全市陆地森林覆盖率达到29.8%。“三优三保”行动全面推进，我市获评全省国土资源节约集约利用模范市。出台生态环境保护工作责任规定，强化环境保护失职追责，环境执法监管水平得到提升。我市获评首批国家生态文明建设示范市，张家港市、常熟市成为国家生态园林城市，苏州工业园区、苏州高新区成为全国首批绿色园区。

　　5．社会事业全面进步，民生福祉持续增进。新建中小学、幼儿园66所，改扩建31所，提档升级外来工子女学校75所，新增学位5.5万个。苏州教育博物馆建成开馆，新增未成年人社会实践体验站112个。在苏高校办学水平全面提升，苏州大学入选国家一流学科建设高校。深化产教融合、校企合作，职业教育加快发展。实施健康城市、健康市民、健康卫士系列行动计划，建立23个医联体、覆盖137家医疗机构，急慢分治的分级诊疗体系基本形成；引进27个国内顶尖临床医学专家团队；市广济医院和第五人民医院建成使用。改扩建15个镇（街道）文化中心，完成422个村（社区）综合性文化服务中心标准化建设任务。加强水乡古镇保护，实施“天堂苏州·百园之城”计划，公布首批濒危非物质文化遗产项目和第三批苏州园林名录，近现代中国苏州丝绸档案入选联合国教科文组织世界记忆名录。《苏州丝绸志》《苏州佛教寺院志》出版发行。“10分钟体育休闲生活圈”加快构建，新增健身步道315公里，人均体育场地面积达到3.3平方米。我市运动员在第十三届全国运动会上取得优异成绩。打造文明城市“升级版”，苏州市实现全国文明城市“四连冠”，张家港市实现“五连冠”，常熟市跻身全国文明城市行列，新增全国文明单位、村镇、校园23家。县级市（区）、镇（街道）、村（社区）社会综合治理三级联动中心全面建立，实现治理网格全覆盖；城乡社区服务社会化试点扩大到85个街道（镇）、284个社区（村）。社会信用体系建设步伐加快，我市综合信用指数继续位居全国地级市首位。严格落实安全生产责任制，深化安全隐患大排查大整治，事故起数、死亡人数实现双下降。推进信访突出问题包案化解，加大矛盾纠纷调处力度，信访总量下降28%。立体化信息化社会治安防控体系逐步完善，治安形势持续向好，张家港市获评全国平安建设先进县（市）。民族团结进步活动广泛开展，宗教工作进一步加强。支持驻苏部队建设，完善优抚安置政策，军政军民团结更加巩固。

　　富民惠民力度加大，民生质量继续提高。城镇居民人均可支配收入达到5.88万元，农村居民人均可支配收入达到2.99万元，分别增长8.1%和8%，38项实事项目全部完成。全市新增就业17.2万人，苏州籍高校毕业生就业率达到98.7%，城镇登记失业率为1.82%。城镇职工社会保险、城乡居民养老保险和医疗保险保持全覆盖，城乡居民基础养老金继续提高，居民医保财政补贴提高到每人每年480元，最低生活保障标准提高到每人每月875元。居家和社区养老服务改革、医养融合、长期护理保险三项国家试点工作积极开展，新增养老机构床位1043张，护理型床位比重达到53.5%。新建公共场所母乳哺育室116家，建设经验在全省推广。建立困难家庭“一户一档一策”精准帮扶机制和低保对象就业成本扣减机制，加大医疗救助力度，落实困难残疾人生活补贴和重度残疾人护理补贴政策，困难群众托底保障网更加牢固。新增缴存住房公积金职工81.1万人，净增6.5%。保障性住房和棚户区（城中村）改造安置住房建设超额完成省下达任务。国家“公交都市”创建工作积极开展，市区新增和优化公交线路55条，新投放公共自行车7900辆。粮安工程三年行动计划如期完成，粮食保供能力得到增强。深入开展食品药品放心行动，食用农产品批发市场销售质量安全监管体系基本建立，食品千人抽检率保持全省第一。物价水平保持稳定，居民消费价格涨幅为1.7%。

　　过去的一年，我们尤为重视政府自身建设，努力打造人民满意的政府。认真学习宣传贯彻党的十九大精神，切实增强政治意识、大局意识、核心意识、看齐意识，始终在思想上政治上行动上同以习近平同志为核心的党中央保持高度一致。不折不扣贯彻中央关于党的建设的一系列重大部署和省委、市委工作要求，强化“一岗双责”，全面提升政府系统党建工作水平。推进“两学一做”学习教育常态化制度化，开展“六个一”基层走访调研和大排查大整治行动，切实改进工作作风，不断密切与人民群众的联系。刚性落实中央八项规定、省委十项规定和市委十二项规定精神，严厉整治“四风”问题，持续推进“清风行动”，严肃查处群众身边的不正之风。厉行勤俭节约，反对铺张浪费，市级“三公”经费下降10%。加强监督执纪问责，坚决防止和查处不作为、慢作为、乱作为等问题。突出挺纪在前，坚持反腐败无禁区、全覆盖、零容忍，用好监督执纪“四种形态”特别是第一种形态，政府系统党风廉政建设全面加强。健全法治政府建设责任制，强化政府及部门主要负责人职责。提请市人大常委会制定地方性法规2件、修改8件、废止1件，颁布政府规章3件，制定规范性文件5件。行政执法和行政复议更加规范，政府信息公开工作不断加强。加大重大政策措施跟踪、重大投资项目、资源环境责任、领导干部经济责任和国有企业审计力度，宽领域、深层次发挥审计监督作用。自觉接受市人大及其常委会法律监督、工作监督和市政协民主监督，坚持重大事项向市人大常委会报告、向市政协通报，注重发挥各民主党派、工商联、无党派人士和人民团体的参政议政作用。组织实施人大议案1件，办复人大代表建议164件、政协提案367件。

　　各位代表，过去的一年，成绩来之不易，值得倍加珍惜。这是以习近平同志为核心的党中央和省委、省政府正确领导的结果，是市委带领全市人民团结奋斗、攻坚克难的结果。在此，我代表市政府向全市人民，向人大代表、政协委员，向离退休老同志，向各民主党派、工商联和无党派人士，向各人民团体，向驻苏解放军、武警和消防官兵，向国家和省驻苏单位，向参与、支持、关心苏州各项事业发展的海内外朋友，表示衷心的感谢和崇高的敬意！

　　在看到成绩的同时，我们也清醒地认识到，经济社会发展和政府工作仍然存在不少问题与薄弱环节，主要是：经济发展质量不够高，新旧动能转换和产业升级步伐不够快；公共服务供给仍显不足，群众在上学、看病、养老、出行等方面还有许多不满意的地方；节能减排、环境保护任务艰巨，让天更蓝、水更清必须久久为功、持续发力；安全生产形势不容乐观，少数领域事故多发频发；城乡之间、区域之间发展不平衡依然存在；创新社会治理、维护公平正义、增进民生福祉还要下更大力气；政府作风效能建设有待加强，工作人员特别是领导干部的能力水平需要进一步提高。对于这些问题，必须采取更为有效措施，努力加以解决。

　　二、以党的十九大精神为指引奋力开辟苏州发展新境界

　　党的十九大对新时代推进中国特色社会主义伟大事业和党的建设新的伟大工程作出了全面部署。习近平新时代中国特色社会主义思想是党的十九大精神的灵魂和主线。贯彻党的十九大精神，必须以这一马克思主义中国化最新成果为行动指南，始终在政治立场、政治方向、政治原则、政治道路上同以习近平同志为核心的党中央保持高度一致，坚定不移忠诚核心、拥戴核心、维护核心、紧跟核心。党的十八大以来，习近平总书记先后三次对江苏工作提出明确要求，我们要以习近平总书记对江苏工作的一系列重要指示为根本遵循，做到坚持和发展中国特色社会主义一以贯之，推进党的建设新的伟大工程一以贯之，增强忧患意识、防范风险挑战一以贯之，坚持系统化思维，注重规律性把握，以更高标准谋划工作，以更大力度狠抓落实，充分展现苏州发展的创新性、探索性、引领性。

　　决胜高水平全面建成小康社会，是当前的紧要任务。我们必须主动对接人民美好生活需要，突出创新驱动、突出改革开放、突出城乡建设、突出文化传承，全力攻克发展不平衡不充分的问题，使发展质量更高、效益更好、更加公平，使人民获得感、幸福感、安全感更加充实、更有保障、更可持续，确保小康路上一个不少、一户不落，确保建成的小康社会得到人民认可、经得起历史检验。创新引领率先实现优化发展，是中央对东部地区提出的明确要求。勇当高水平全面建成小康社会的标杆，勇当探索具有时代特征、江苏特点的中国特色社会主义现代化道路的标杆，是省委赋予苏州的光荣使命，也是苏州自身发展的客观要求和全市人民的热切期盼。我们有条件也有责任率先推动高质量发展，加快建设具有国际竞争力的现代产业名城、开放包容的创新创业名城、富裕文明的美丽宜居名城、古今辉映的历史文化名城，争做“强富美高”新江苏建设先行军排头兵。

　　在今后工作中，我们将切实把握以下五点：

　　1．推动高质量发展，必须加快培育经济发展新动能。深刻领会、坚决贯彻习近平新时代中国特色社会主义经济思想，践行新发展理念，努力建设现代化经济体系。以供给侧结构性改革为主线，推动经济发展质量变革、效率变革、动力变革，加快构建实体经济、科技创新、现代金融、人力资源协同发展的产业体系。坚持把创新作为引领发展的第一动力，强化科技创新的战略支撑作用，优化创新生态系统，集聚高端创新要素，重点围绕转型升级，努力实现引领性原创成果重大突破，加快形成创新高峰，打造具有全球影响力的产业科技创新高地。倡导创新文化，激发和保护企业家精神，弘扬劳模精神和工匠精神，营造大众创业、万众创新的浓厚氛围。坚持高端化、智能化、绿色化、服务化、品牌化发展方向，突出质量强市，建设“中国制造2025”国家级示范区，打造具有国际竞争力的先进制造业基地。大力发展现代服务业，推动生产性服务业向专业化和价值链高端延伸，生活性服务业向精细化和高品质方向提升。实施乡村振兴战略，构建现代农业产业体系、生产体系、经营体系，建设美丽宜居乡村，推进农业农村现代化。

　　2．推动高质量发展，必须更好满足民生幸福新期盼。坚持以人民为中心，坚持人人尽责、人人享有，坚守底线、突出重点、完善制度、引导预期，推进基本公共服务均等化、标准化，着力在幼有所育、学有所教、劳有所得、病有所医、老有所养、住有所居、弱有所扶上取得新进展，促进人的全面发展、全市人民共同富裕。贯彻就业优先战略和积极就业政策，以创业带动就业，实现更高质量和更充分就业，持续增加居民收入。加快教育现代化，办好人民满意的教育。落实“健康苏州2030”规划，实施卫生资源补短补缺计划。积极应对人口老龄化，推进老龄事业和产业发展。坚持兜底线、织密网、建机制，努力形成覆盖全民、城乡统筹、权责清晰、保障适度、可持续的多层次社会保障体系。统筹城乡社会救助体系，完善最低生活保障制度。加快建立多主体供给、多渠道保障、租购并举的住房制度。打造共建共治共享的社会治理格局，提高社会化、法治化、智能化、专业化水平。

　　3．推动高质量发展，必须持续增创改革开放新优势。坚决破除一切不合时宜的思想观念和体制机制弊端，为高质量发展增添强大动力。进一步转变政府职能，深化简政放权，创新监管方式，构建“亲”“清”政商关系，营造更加透明、便利、公平的市场环境和营商环境。清理废除妨碍统一市场和公平竞争的各种规定和做法，支持民营企业发展，激发各类市场主体活力。完善产权制度和要素市场化配置，实现产权有效激励、要素自由流动、价格反应灵活、竞争公平有序、企业优胜劣汰。深度融入全球经济体系，发展更高层次的开放型经济。推进贸易和投资自由化便利化，复制推广自由贸易试验区和苏州工业园区改革经验，主动对接自由贸易港政策。实施准入前国民待遇加负面清单管理制度，放宽市场准入，扩大服务业对外开放。深入推进苏州工业园区国家开放创新综合试验，积极探索海峡两岸产业合作新路径新模式，打造中德合作典范城市。创新对外投资方式，以“一带一路”为重点，扩大国际产能合作，加强境外资源开发，构建面向全球的贸易、投融资、生产、服务网络。更好发挥苏州在长江经济带、长三角世界级城市群和扬子江城市群建设中的积极作用，提升城市竞争力和可持续发展能力。

　　4．推动高质量发展，必须全力打造美丽宜居新家园。秉持“绿水青山就是金山银山”的理念，更大力度加强生态文明建设，努力形成节约资源和保护环境的空间格局、产业结构、生产方式、生活方式，促进人与自然和谐共生。优化生态文明体制机制，健全生态补偿机制、生态环境监管及责任追究制度。实施生态保护和修复重大工程，建设生态廊道和生物多样性保护网络，守牢生态保护红线、永久基本农田、城镇开发边界三条控制线，提升生态系统质量和稳定性。坚持绿色发展，力促资源能源消耗明显下降、污染排放持续削减，建设国际能源变革典范城市。倡导简约适度、绿色低碳的生活方式，反对奢侈浪费和不合理消费。匠心打磨城市建设的内核细节，注重延续城市肌理和水乡韵味，凸显古城粉墙黛瓦、小桥流水的独特城市标签和城市记忆，绘好现代版的“姑苏繁华图”。

　　5．推动高质量发展，必须精心谱写文化兴盛新篇章。坚持中国特色社会主义文化发展道路，弘扬优秀传统文化，推动创造性转化、创新性发展，努力把苏州建成世界级历史文化名城。切实保护文化遗存，丰富拓展文化内涵，让厚重历史感和鲜明时代感有机相融，真正做到留住历史记忆、彰显时代价值。优化公共文化服务，强化精准供给、多样供给、品质供给、特色供给，使公共文化服务更加贴近群众需要。深化文化体制改革，完善文化经济政策，创新生产经营机制，培育新型文化业态，在社会效益优先基础上，实现文化产业社会效益和经济效益双丰收。坚持以人民为中心的创作导向，增强文艺原创力，多出思想精深、艺术精湛、制作精良的精品力作。坚定文化自信，加强人文交流，以我为主、兼收并蓄，加快文化走出去步伐，向世界传播苏州声音、讲好苏州故事，提升苏州文化的国际影响力。

　　三、2018年主要任务

　　2018年是贯彻党的十九大精神的开局之年，是改革开放40周年，是决胜高水平全面建成小康社会、实施“十三五”规划承上启下的关键一年。根据中共苏州市委十二届五次全会的部署，今年政府工作的总体要求是：全面贯彻党的十九大精神，以习近平新时代中国特色社会主义思想为指导，深入落实习近平总书记视察江苏重要讲话精神，坚持稳中求进工作总基调，坚持新发展理念，紧扣社会主要矛盾变化，按照高质量发展的要求，统筹推进“五位一体”总体布局，协调推进“四个全面”战略布局，以供给侧结构性改革为主线，以勇当“两个标杆”为方向，以落实“四个突出”为重点，以建设“四个名城”为目标，以实施十二项“三年行动计划”为主要抓手，全力做好改革发展稳定各项工作，推动经济健康发展、社会和谐进步。

　　全市经济社会发展的主要预期目标是：地区生产总值增长7%左右，一般公共预算收入增长8%以上，全社会固定资产投资与上年持平，社会消费品零售总额增长8%，进出口总额保持稳定，服务业增加值占地区生产总值比重提高0.5个百分点以上，全社会研究与试验发展经费支出占地区生产总值比重达到2.9%左右，企业研究与试验发展经费投入增长10%左右，万人有效发明专利拥有量50件以上，全员劳动生产率增长7%以上，工业增加值率提高0.5个百分点左右，居民人均可支配收入增长高于地区生产总值增幅，居民消费价格总水平涨幅控制在3%左右，城镇登记失业率控制在3%以内，单位地区生产总值能耗下降、主要污染物排放总量削减完成省下达任务。

　　在新的一年里，我们将重点做好八个方面工作：

　　1．着力促进经济提质增效。以提高供给体系质量为主攻方向，推动产业迈向中高端水平。一是做大做强实体经济。把发展经济的着力点放在实体经济上，强化政策支持，优化发展环境，营造崇尚实业的社会氛围。加强政府服务和要素保障，提升财税金融服务实体经济能力。以制造业为重点，引导企业加大投入、加强研发、加快发展，打造产品质量高、市场占有率高、品牌知名度高的标志性龙头企业，培育“专精特新”中小企业，形成大中小企业专业化分工协作的生动局面。二是大力发展新兴产业。重点布局新一代信息技术、生物医药、纳米技术、人工智能、物联网等先导产业，每个县级市、区精准培育1～2个先导产业，全市新兴产业产值占规模以上工业总产值比重提高1个百分点。三是改造提升传统产业。开展“千企技改升级”“千企小升规”和质量品牌提升行动，提高产品技术、工艺装备、能效环保水平，技改投资占工业投资比重保持在70%左右。推进智能制造，发展服务型制造，创建30家以上省级示范智能车间，新增10家省级服务型制造示范企业。推进工业企业资源集约利用，实施差别化价格政策，倒逼企业转型发展。推广绿色建材和装配式建筑。四是加快发展现代服务业。做大做强生产性服务业，发展新型生活性服务业。建设大数据产业园，发展数字经济、平台经济，举办全球人工智能产品应用博览会。以工业软件为特色，争创中国软件名城。以新兴产业和高端服务业为重点，建设特色小镇。五是全面做好“破”“立”“降”工作。推动互联网、大数据、人工智能和实体经济深度融合，在中高端消费、创新引领、绿色低碳、共享经济、现代供应链、人力资本服务等领域培育新增长点、形成新动能。大力破除无效供给，继续关停淘汰落后低效企业，完成省下达的各项去产能任务，减少商业办公用房库存。多措并举降低实体经济成本，降低制度性交易成本。加强地方政府债务管理，降低企业杠杆率，防范和化解区域性金融风险。精心开展第四次全国经济普查。

　　积极扩大有效需求，促进经济平稳增长。一是优化投资结构。建设210项重大项目，年内完成投资1300亿元左右。大力推进14个单项投资百亿元以上的产业项目，加快实施补短板项目。全年服务业投资占全社会固定资产投资比重超过60%；完成工业投资1500亿元左右，新兴产业投资占到68%。二是培育消费热点。鼓励体验消费、智能消费、定制消费等新型消费，促进消费升级。保护“老字号”品牌，加快传统商贸业转型发展。丰富旅游业态，优化旅游服务，注重发展精品特色住宿和夜旅游产品，创建全国全域旅游示范区。推进文化消费试点，挖掘文化消费资源，推动文旅深度融合，引导居民扩大文化消费。三是拓展对外贸易。深耕传统市场，开拓新兴市场，提高与“一带一路”沿线国家和地区的贸易比重。扩大全球维修、一般纳税人资格等试点范围，引导具备条件的加工贸易企业向全产业链、自主品牌、内外贸一体化和总部经济方向转型。创新发展服务贸易，力争服务贸易增长10%。加快跨境电子商务综合试验区建设，跨境电商、市场采购贸易、外贸综合服务等新业态出口增长10%。建设开放创新国检综合试验区。高水平办好中国（昆山）品牌产品进口交易会。

　　2．着力加快科技创新步伐。完善区域创新体系，用好优质创新资源，增强创新驱动能力。一是建设重大创新载体。更好发挥在苏高校作用，更大力度吸引国内外科研院所、知名高校和龙头企业来苏共建研发机构，集中力量攻克核心技术、共性技术，促进产业转型升级。二是打造创新企业集群。实施高新技术企业培育计划，新增国家高新技术企业500家、省级民营科技企业1000家。支持有条件的企业设立先进技术研究院，参与建设国家级、省级创新中心，争创国家智能人机交互产业创新中心。三是集聚创新创业人才。实施人才引育工程，发挥“一站式”服务平台作用，新增高层次人才2.25万人、高技能人才2万人。办好苏州国际精英创业周、海外创业大赛苏州总决赛。四是深化科技金融结合。扩大科技金融风险补偿范围，实现“科贷通”贷款75亿元。做大天使投资引导基金，鼓励创投机构发展，引导更多社会资本投向新兴产业和初创期科技企业。五是提升科技服务水平。开展企业研发费用加计扣除政策落实专项行动，降低企业创新成本。完善国家技术转移苏南中心功能，促进资源共享、技术转移和成果转化。争取设立省药品医疗器械审评分中心。强化知识产权创造、保护、运用，实施企业知识产权登峰行动计划，健全知识产权运营服务体系，办好第七届中国国际版权博览会。推动众创空间发展，建设“运河湾”创新创业中心。

　　3．着力增强发展动力活力。聚焦重点领域和关键环节，推动改革全面发力、多点突破。一是深化行政体制改革。推进相对集中行政许可权改革试点，调整完善权力清单，提升政务服务网建设水平，推动“网上办、不见面”，实现高效审批。加快综合行政执法体制改革，在有条件的区域、领域实行“一支队伍管执法”。做好经济发达镇行政管理体制改革试点工作。二是深化投融资体制改革。用好城市发展基金、创新产业发展引导基金，发挥政府投资引导和带动作用。推行政府和社会资本合作模式，鼓励社会资本投向基础设施、生态环保和社会建设等领域。三是深化商事制度改革。推行登记信用承诺制度和全程电子化登记，开展“证照分离”改革，推进“多证合一”，促进市场准入便利化。四是深化财税金融改革。加强财政资金绩效管理，压缩一般性支出。推进国税地税征管体制改革综合试点。开展资源税改革，落实环境保护“费改税”政策措施。完善综合金融服务平台功能，更好满足企业融资需求。争取设立昆山两岸金融创新合作综合改革试验区。五是深化国资国企改革。完善国有资产管理体制，推动国有资本向重要行业、关键领域、重点基础设施、前瞻性战略产业集中，促进国有资产保值增值，做强做优做大国有资本。推动国有企业完善现代企业制度，健全公司法人治理结构，开展外派董事试点，探索推行职业经理人制度，发展混合所有制经济。

　　加强开放合作，厚植开放型经济优势。一是加大引进来力度。营造内外资一致的市场环境，稳定使用外资规模，提高使用外资水平。更加注重高端制造业和现代服务业领域的招商引资，新兴产业和高技术项目实际使用外资比重超过50%。鼓励境外投资者以并购、境内股权出资、跨境人民币出资、无形资产出资等方式投资，支持本土企业通过境外上市、发行债券等方式拓宽使用外资渠道。二是加快走出去步伐。培育壮大跨国经营主体，支持企业加强海外并购和市场开拓。推进埃塞俄比亚东方工业园二期工程，争取印度尼西亚吉打邦农林生态产业园成为省级境外经贸合作产业集聚区。建设“一带一路”苏州国际铁路物流中心，推动中欧（苏州）班列、“苏新亚”班列、太仓港长江水运集散中心持续发展。做好外事、对台和侨务工作，促进经贸合作与人文交流，建好布达佩斯中国文化中心。抓好挂钩共建、对口支援和协作帮扶工作。三是推动开发区创新发展。贯彻促进开发区改革发展的政策措施，落实国家级开发区全链审批赋权清单，打造特色创新集群，提升土地产出率、资源循环利用率和智能制造普及率。推进开发区区域能评、环评改革，创建绿色低碳生态园区。

　　4．着力推进城乡融合发展。健全城乡融合发展体制机制和政策体系，提升城乡一体化水平。一是精心编制各类城乡规划。抓紧编制苏州市城市总体规划（2035），高度重视优化城市功能，提升城市能级，确保年内上报国务院。抓好城市设计试点工作，实现古城城市设计全覆盖。推动各镇总体规划修编，优化重点村、特色村规划。建设统一开放的规划信息平台，促进“多规合一”。二是加强城乡基础设施建设。加快建设沪通铁路苏州段，力争开工建设通苏嘉、沪苏湖、太仓港港口支线和苏南沿江铁路。推进太仓港集装箱码头四期工程建设，启动张家港港区集装箱码头改扩建工程。实施国际物流快速通道二期、东环南延、桐泾路北延、城北路改造、金鸡湖隧道、独墅湖第二通道等工程，改扩建312国道苏州东段和524国道相城段。继续建设轨道交通3号线、5号线，力争开工S1号线和6号线、8号线。做好地下综合管廊国家试点项目的管线入廊及运行维护工作，建成海绵城市试点项目。推进长江堤防防洪能力提升、京杭大运河堤防加固、东太湖综合整治续建、阳澄湖中西湖连通等工程。高标准完成重要经济目标防护建设国家试点任务，新增人防设施100万平方米。做好5G通信试点工作，建设“宽带中国”示范城市。推动农村基础设施提档升级，优化提升建制镇，整治提升被撤并镇。三是提高城乡管理水平。持续开展城市环境综合整治，建成智慧城管大数据平台，完善常态长效管理机制。综合整治老旧小区，治理改造城中村（无地队），清理违法建设。大力推进历史文化名城保护和提升六大工程，继续建设虎丘地区综合改造、桃花坞片区综合整治保护利用和“两河一江”综合整治项目，实施山塘历史街区风貌整治工程，做好整体保护与有机更新、特色塑造与品质提升、环境治理与设施配套、民生改善与社会和谐、业态转型与文化兴盛五篇文章，增强古城魅力与活力。加强农村突出环境问题综合治理，推进美丽乡村建设，抓好5个特色田园乡村省级试点和20个市级试点，建设10个康居特色村和350个三星级康居乡村，传承发展提升农耕文明。

　　按照产业兴旺、生态宜居、乡风文明、治理有效、生活富裕的总要求，加快乡村振兴，推动农业全面升级、农村全面进步、农民全面发展。一是加快发展现代农业。培育新型农业经营主体和社会化服务主体，发展多种形式适度规模经营，促进农村一二三产业融合发展。推进农业供给侧结构性改革，坚持质量兴农、绿色兴农，优化“四个百万亩”布局，发展高效设施农业和“互联网+现代农业”，新增现代农业园区4万亩、高标准农田5万亩、高标准池塘养殖面积6.5万亩，做大特色产业，做优特色品牌，提高农业创新力、竞争力和全要素生产率。二是深化城乡一体化改革。强化乡村振兴制度性供给，抓好国家级农村改革试点工作。深化农村集体产权制度改革，完善承包地“三权分置”制度。加大公共财政向“三农”倾斜力度，提升金融服务乡村振兴能力和水平，引导资本、人才、技术等要素向农村流动，鼓励社会力量参与乡村建设。加强农村基层基础工作，培养造就懂农业、爱农村、爱农民的“三农”工作队伍。三是壮大农村集体经济。发展股份合作经济，建设富民强村载体，加强对经济薄弱村的政策扶持，增加村均稳定性收入。基本完成农村集体资产清产核资，建成农村集体“三资”监管信息系统，保障农民财产权益。

　　5．着力加强生态文明建设。贯彻节约优先、保护优先、自然恢复为主的方针，扎实开展“两减六治三提升”专项行动，持续提高生态环境质量。一是坚决完成省下达的“减煤”“减化”任务。落实节能减排措施，开展锅炉整治、产能压缩、集中供热和散煤治理，健全耗煤项目准入和淘汰机制，降低煤炭消费总量。深入实施化工行业优化提升整治专项行动，关停淘汰落后化工企业。二是打好气、水、土治理攻坚战。实施重点行业提标改造、挥发性有机物治理、机动车尾气治理、扬尘整治等大气治理项目，确保PM2.5浓度和优良天数比例达到省考核要求。落实河（湖）长制和断面长制，做好治河（湖）、护河（湖）工作，推进太湖流域和金鸡湖水环境综合治理，完成阳澄湖新三年生态优化行动计划，基本完成太湖4.5万亩围网养殖清理任务，治理非禁养区规模畜禽养殖场，继续整治黑臭河道，推动水质持续向好。落实土壤污染防治工作方案，加强污染状况调查与治理修复。三是强化生活垃圾和固体废弃物处置。新增一批生活垃圾分类试点，实现市区公共机构和相关企业生活垃圾强制分类，城乡生活垃圾分类投放设施覆盖率达到60%。开工建设七子山静脉产业园焚烧发电提标改造项目。新建改建公共卫生间250座。建设固体废弃物处置设施，促进无害化处理、资源化利用。推进危险废物去库存、减量化。四是加强生态建设与保护。持续开展绿化造林，建设生态公园，完成森林抚育4万亩，市区新增及改造绿地350万平方米。加强湿地保护，出台县级市、区湿地保护规划。严格保护耕地，实施轮作休耕。挖掘存量土地潜力，促进低效用地再开发。开展第三次全国土地调查。五是严格生态环境监管。落实生态环境保护工作责任和生态文明建设领域失职追责制度，全面推行领导干部自然资源资产离任审计，配合国家审计署做好长江经济带生态环境保护审计工作。建立以排污许可证为核心的环境监管新模式，完善环保失信企业联合惩戒机制。深入开展企业环境安全隐患排查治理，继续整治“散乱污”企业，坚决打击环境违法行为。完成环保机构监测监察执法垂直管理制度改革。

　　6．着力提高人民生活质量。坚持从人民群众关心、让人民群众满意的事情做起，办好各项民生实事。一是提高就业质量和居民收入水平。积极扩大就业，城镇新增就业15万人，苏州籍高校毕业生就业率保持在95%以上，零就业、零转移家庭实现动态清零。完善协商协调机制，构建和谐劳动关系。提高创业成功率，推动创业带动就业。支持农民就业创业，拓宽农民增收渠道。二是加强社会保障体系建设。深入推进全民参保登记工作，实施精准扩面。全面实行大病保险制度，扩大长期护理保险试点范围。推行精准帮扶、阳光扶贫，提高最低生活保障标准，加大对因病致贫等困难家庭的帮扶救助力度，发挥慈善事业扶贫济困的积极作用。扩大养老服务供给，构建多元养老服务模式。实施镇（街道）敬老院改造升级三年行动计划，增加养老机构床位、日间照料中心和助浴点。推动医养融合发展，推广家庭医生签约服务。贯彻男女平等基本国策，保障妇女儿童合法权益。加强残疾康复服务，提高残疾人保障水平。三是改善群众生活条件。完善促进房地产市场平稳健康发展的长效机制，合理引导市场预期和购房行为。发展住房租赁市场特别是长期租赁，增加保障性住房供给，满足不同群体的住房需求。优化公交线网，增加公交车和公共自行车投放，建成国家“公交都市”。实施优质粮食工程，建设粮食科技创新示范市。改造提升农贸市场，推动农贸市场纳入信息化监管。推进“智慧食药监”工程和食品药品放心行动，建设食用农产品安全风险防控体系，保障人民群众饮食用药安全。加强价格监管和调控，保持价格总水平基本稳定。

　　7．着力推动社会和谐进步。优化公共服务供给，提升社会治理水平，让改革发展成果更多更公平惠及全市人民。一是促进教育优质均衡发展。新建和改扩建中小学24所、幼儿园12所，推进义务教育学校标准化建设。加强校园安全防范，提高校园安保水平。完善现代职业教育和培训体系，创建高水平职业院校。深化名城名校融合发展，促进在苏高校内涵提升。办好继续教育，建设学习型社会。培养高素质教师队伍，倡导全社会尊师重教。二是推进“健康苏州”建设。深化医药卫生体制改革，完善医疗卫生服务体系，新建和改扩建24家基层卫生服务机构，启动市疾病预防控制中心迁建工程。建成市第九人民医院和独墅湖医院，建设苏州大学附属第一医院总院二期和市中医院二期工程，传承发展吴门医派。倡导健康文明生活方式，推动全民健身和全民健康深度融合。建成苏州奥林匹克体育中心、金阊体育馆、东园体育休闲公园，建设运河体育主题公园、东太湖百里健身休闲带和健身步道等设施。三是推动文化繁荣发展。深化党的十九大精神宣传教育，推动习近平新时代中国特色社会主义思想深入人心。加强新型智库建设，发展哲学社会科学。支持文艺创作，推出更多优秀作品。建设大运河文化带，振兴传统工艺美术，打造博物馆城、遗产城市、手工艺与民间艺术之都。实施文化惠民工程，充分利用公共文化设施，丰富群众文化生活。建成苏州第二图书馆，建设苏州第二博物馆、考古博物馆、科技馆（工业展览馆）、艺术大剧院和中国丝绸档案馆。办好第七届中国昆剧艺术节、中国苏州评弹艺术节和第三届世界遗产城市亚太地区大会。做好年鉴和地方志编修工作。四是加强和创新社会治理。深化群众性精神文明创建活动，推动文明城市创建成果常态长效。把培育和践行社会主义核心价值观融入社会发展各方面，转化为市民的情感认同和行为习惯。弘扬科学精神，普及科学知识。巩固全国社会信用体系建设示范城市创建成果，铸造苏州信用品牌。强化顶层设计，建设城市数据大脑，推动政务数据整合共享开放。推行全科社工、智慧社区、协商共治、社区服务社会化、志愿服务制度化等创新举措，完善社会综合治理网格化联动机制，形成治理重心下移、服务扁平高效、各方力量参与的城乡社区治理与服务新格局。落实信访工作责任制，健全矛盾纠纷化解机制，依法及时就地解决群众合理诉求。树立安全发展理念，健全公共安全体系，严格落实安全生产责任制，加大监督检查和失职追责力度，坚决杜绝重特大安全事故，提升防灾减灾救灾和应急处置能力。加强社会治安防控，严密防范、依法打击各类违法犯罪行为，维护国家安全和社会稳定。深化法治城市创建，广泛普及法律知识，健全公共法律服务体系，扩大法律援助范围。深化民族团结进步教育，精心做好宗教工作。完善国防动员体系，加强全民国防教育，落实拥军优属政策，推动军民融合深度发展。

　　8．着力打造为民清廉政府。自觉用习近平新时代中国特色社会主义思想武装头脑、指导实践、推动工作，牢固树立“四个意识”，始终坚定“四个自信”，坚决落实全面从严治党要求，切实做到信念过硬、政治过硬、责任过硬、能力过硬、作风过硬。一是牢记为民宗旨。始终把人民对美好生活的向往作为一切工作的出发点和落脚点，增强服务意识，提高服务效能。推进“两学一做”学习教育常态化制度化，开展“不忘初心、牢记使命”主题教育，促使党员干部做共产主义远大理想和中国特色社会主义共同理想的坚定信仰者和忠实实践者。注重培养政府工作人员的专业精神、专业能力，提高履职本领。二是切实改进作风。为政贵在行。大力弘扬求真务实作风，以钉钉子精神抓好工作落实。坚决贯彻中央八项规定实施细则我市实施办法，深入整治“四风”问题的新表现，克服形式主义、官僚主义，祛除享乐主义、奢靡之风，坚决打好作风建设持久战。大兴调查研究之风，深化“六个一”基层走访调研活动，切实解决群众和企业反映的重点难点问题。不断优化亲商服务，保护、关爱、支持企业家创新创业。健全激励机制和容错纠错机制，更好保护和调动干部干事创业积极性。三是依法履行职责。自觉尊法学法守法用法，坚持做到依法行政。严格执行重大行政决策程序规定，确保决策制度科学、程序正当、过程公开、责任明确。坚决落实市人大及其常委会决议决定，自觉接受人大法律监督、工作监督和政协民主监督，主动听取民主党派、人民团体和社会各界意见建议，提高人大议案、代表建议、政协提案办理质量和办成率。落实合法性审查和公平竞争审查制度，做好行政复议案件办理、行政诉讼案件出庭应诉等工作。深化政务公开，加强信息发布和政策解读，及时准确回应社会关切。四是加强反腐倡廉。强化政府系统党风廉政建设，切实履行“一岗双责”。坚持把纪律和规矩挺在前面，严格纪律执行，让党员干部知敬畏、存戒惧、守底线。加大整治群众身边腐败问题力度，狠抓工程建设、项目审批、资源开发、产权交易、医用耗材采购、扶贫惠农等领域的反腐倡廉工作，强化不敢腐的威慑，扎牢不能腐的笼子，增强不想腐的自觉。倡导勤俭节约，建设节约型机关。加强组织监督、审计监督和日常管理监督，接受司法监督、群众监督和舆论监督，完善绩效管理，努力实现干部清正、政府清廉、政治清明。

　　各位代表，新时代开启新征程，新使命呼唤新作为。让我们更加紧密地团结在以习近平同志为核心的党中央周围，以习近平新时代中国特色社会主义思想为指导，深入贯彻党的十九大精神和习近平总书记视察江苏重要讲话精神，不忘初心，牢记使命，在中共苏州市委的坚强领导下，紧紧依靠全市人民，锐意进取，埋头苦干，为勇当“两个标杆”、争做“强富美高”新江苏建设先行军排头兵而努力奋斗！
